# Supplementary material for: Role of CB1 Cannabinoid Receptors in Vascular Responses and Vascular Remodeling of the Aorta in Female Mice
Source: Int J Mol Sci. 2023 Nov 17;24(22):16429. doi: 10.3390/ijms242216429 (PMC10671338; doi:10.3390/ijms242216429)
Supplement: Supplementary file 1 [file ijms-24-16429-s001.zip › ijms-2635486-supplementary.pdf]

Supplementary Material

S1. Supplementary Methods

S1.1. Determination of estrogen and estrogen metabolite levels by HPLC mass spectrometry

The analysis of the estrone, estradiol, 2-hydroxyestrone, 2-hydroxyestradiol, 4-hydroxyestrone and 4-hydroxyestradiol was performed at the Laboratory of Mass Spectrometry and Separation Technology, Department of Laboratory Medicine, Semmelweis University (Budapest, Hungary) using an in-house method. Two aliquots of plasma, each with a volume of 200 μL, were prepared for analysis. Aliquots with a volume less than 200 μL were diluted using estrogen-stripped liquid chromatography-mass spectrometry grade water as necessary. 10 μL internal standard solution containing the isotopically labeled analogues of the analytes was spiked to each aliquot. To the first aliquot 0.2 mL of an aqueous solution of β-glucoronidase/aryl sulfatase was added. The mixture was kept at 60 °C for 120 min, followed by centrifugation and dilution with 0.2 mL water containing 0.1% formic acid. The second aliquot was diluted with 0.2 mL water containing 0.1% formic acid, and was not treated with the β-glucoronidase/aryl sulfatase solution. Both aliquots underwent simplified liquid extraction and derivatisation with 2 mg/mL dansyl chloride in acetonitrile. Analysis was performed using two-dimensional ultra-high performance liquid chromatography [using a Phenomenex Kinetex XB-C18 and a Kinetex Biphenyl stationary phase (Gen-Lab Kft., Budapest, Hungary)] coupled with triple-quadrupole mass spectrometry. The mass spectrometer was operated in the multiple reaction monitoring mode following positive electrospray ionization. The precursor ions were the $[M+H]^+$ ions of the dansylated analytes, while the product ions were m/z=171. For quantitation, plasma calibrators and linear calibration models with $1/x^2$ weights were employed. The plasma calibrators were made of charcoal-stripped human plasma left over from clinical laboratory tests performed at the Department of Laboratory Medicine, Semmelweis University (Budapest, Hungary). De-identification of the plasma samples had been performed before using them for research.

S1.2. Myography to detect the role of constrictor prostanoids in acetylcholine-induced vasorelaxation of aorta of female CB_1R knockout (CB_1R-/-) and wild-type (CB_1R+/+) mice

Additional experiments on myography was performed (see Methods 4.2, 4.3) with a specific inhibitor of thromboxane-prostanoid (TP) receptors SQ 29,548 ((5Z)-7-[(1S,2R,3R,4R)-3-[[2-[(phenylamino)carbonyl]hydrazinyl]methyl]-7-oxabicyclo[2.2.1]hept-2-yl]-5-heptenoicacid, 10^{-6} mol/L, Cayman Chemical, Ann Arbor, MI USA) to detect the role of constrictor prostanoids (such as TXA_2, PGH_2) [1] in acetylcholine-induced vasorelaxation of aorta of female CB_1R KO (CB_1R−/−) and wild-type (CB_1R+/+) mice. Experiments were performed on abdominal aortas of female CB_1R KO and wild-type mice (20-23 g, n=4) as described in 4.3. TP receptor inhibitor SQ 29,548 or vehicle was added to the baths for 30 minutes prior to agonist-induced responses. Dose-response curves to acetylcholine (Ach, 10^{-8}-10^{-6} mol/L) was obtained after phenylephrine-induced precontraction (10^{-5} mol/L). Relaxation responses were calculated as percent values

from precontraction level (see Methods 4.6.) and statistical analysis was performed with pairwise comparisons by ANOVA. Significance level was set to p<0.05.

S1.3. Immunohistochemistry to detect levels of thromboxane synthase enzyme protein in aorta of female CB_1R knockout (CB_1R-/-) and wild-type (CB_1R+/+) mice

We have made immunostaining to detect thromboxane synthase according to our previous protocols as described (Section 4.5 in Methods) we use the following methods: deparaffinisation of 7μm thick paraffin embedded sections were made by citrate puffer (pH=6) based antigen retrieval. Endogenus peroxidase activity was blocked with 3% H_2O_2. To eliminate the nonspecific labelling of the secondary antibody we used a 2.5% normal horse serum (NHS) blocking solution (Vector Biolabs, Burlingame, CA, USA). Mouse monoclonal TBXAS primary antibody (Invitrogen – Thermo Fischer Waltham, Massachusetts, USA) was used with overnight application at 4°C in 1:50 concentration. For secondary labeling we used horseradish-peroxidase-(HRP) linked anti-mouse polyclonal antibody (Vector Biolabs, Burlingame, CA, USA). Visualization was performed by brown colored horse radish peroxydase (HRP) linked 3′3 – diaminobenzidine (DAB, Vector Biolabs, Burlingame, CA, USA). For counterstaining we used purple colored hematoxylin (Hematoxylin modified to Gill II, Sigma-Aldrich, St. Louis Missouri USA). After covering slides were photographed with Nikon eclipse Ni-U microscope with DS-Ri2 camera (Nikon, Minato - Tokyo Japan). Photos were taken at 20x magnification, The evaluation measured the optical densitometry of the brown color with FIJI® software package (NIH Bethesda, MA, USA).

S1.4. Pharmacological analysis of inhibitor responses

Pharmacological analysis of inhibitor responses described in Results section 2.2 was performed by the analysis of Emax and EC50 values obtained from dose-response curves (Supplementary Table 1).

S2. Supplementary Results

Inhibitor of TP receptors SQ 29,548 in CB_1R+/+ wild-type aortas augmented Ach-induced relaxation similarly to the COX-inhibitor indomethacin (Suppl. Fig. 1A, Fig. 2C, p<0.05 at 10^{-6} mol/L) which effect was not observed in CB_1R KO mice (Suppl. Fig. 1B, Fig. 2D). These results indicate that arachidonic acid (AA) metabolites -which induce constrictor responses mostly via TP receptors- modulate Ach-induced vasorelaxation in WT, but not in CB_1R KO mice.

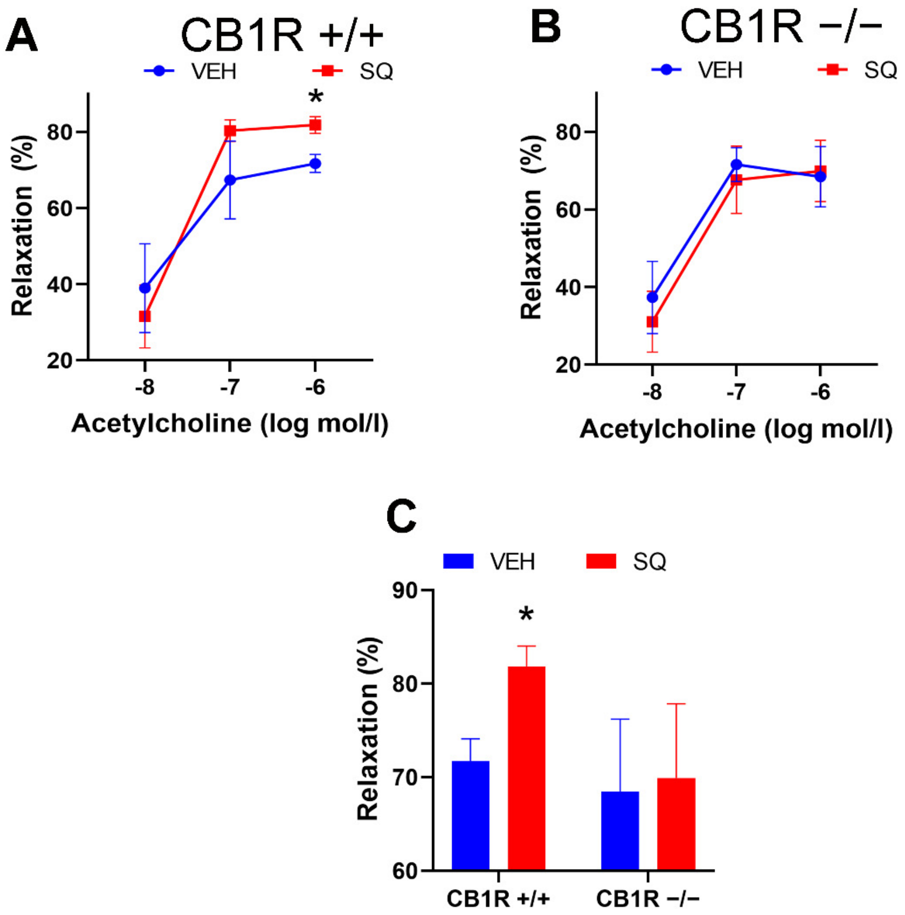

Supplementary Figure S1. Effects of specific inhibitor SQ 29,548 (SQ, inhibitor of thromboxane-prostanoid receptors) in aortas of wild-type (WT, CB1R+/+) and CB1R KO (CB1R−/−) female mice. **Panel A-B**. Effects of inhibitor on acetylcholine (Ach)-induced vasodilation of aortas from CB1R+/+ and WT female mice (n=4) precontracted with phenylephrine. **Panel C**. Effects of inhibitor on Ach-induced vasodilation at 1 μmol/L Ach-concentration. Data are shown as Mean ± SEM values. P<0.05 values were considered significant. *: p<0.05, between vehicle (VEH) and SQ-treated segments, (Relaxation data were calculated as percent values of precontraction level.)

Immunohistochemical staining was performed with thromboxane synthase (TXBAS) to detect the role of constrictor metabolites in the aorta of CB_1R KO (CB1−/−) and wild-type (CB1+/+) mice. We have found that the level of TXBAS had a tendency to increase in the wild-type aortas compared to CB_1R KO which did not reach the statistical significancy (p=0.06, unpaired t test).

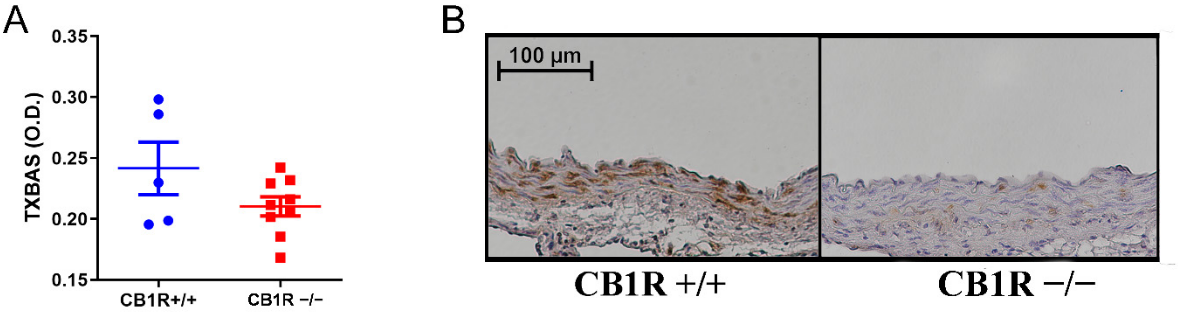

Supplementary Figure S2. Panel A. Results of immunohistochemical staining of thromboxane synthase (TXBAS) on aortas of wild-type (CB1R+/+, n=5) and CB1R KO (CB1R−/−, n=9) mice. **Panel B.** Representative photos of TXBAS immunostained aorta segments, visualization with DAB on hematoxylin counterstaining, photographed at 20x magnification. Evaluation was performed from the values of the media layer. Statistical analysis performed with unpaired t-test. Data shown by non-calibrated optical density with mean ± SEM Abbreviations: CB1R: cannabinoid type 1 receptor, CB1R+/+: cannabinoid type 1 receptor wild-type mice, CB1R−/−: cannabinoid type 1 receptor knockout mice, DAB: diamino-benzidine, O.D.: optical density.

Results from pharmacological analysis of inhibitor responses (see also Results section 2.2) with analysis of effective maximum value (Emax), effective concentration at 50% of maximum value (EC50) and negative log of effective concentration at 50% (pEC50) values obtained from dose-response curves are indicated in Supplementary Table 1.

Supplementary Table S1. Pharmacological analysis of data from myograph with inhibitors Nω-nitro-L-arginine (LNA, inhibitor of nitric oxide synthase) and indomethacin (INDO, inhibitor of cyclooxygenase) or vehiculum (veh) on contraction-relaxation vascular responses in aortas of wild-type (CB1R +/+) and CB1R knockout (CB1R −/−) female mice (analysis from data of Figure 2). Emax %, EC50 and pEC50 values are indicated. Mean ± SE values. CB1R: cannabinoid type 1 receptor, Emax: effective maximum value, EC50: effective concentration at 50% of maximum value, pEC50: negative log of effective concentration at 50%, SE: standard error

VEHICULUM

genotype	treatment	N value	Emax%	SE(Emax%)	EC50 nmol/l	SE(EC50)	pEC50
CB1 +/+	Ach veh	8	75,3	2,70	18,80	4,20	7,72
CB1 −/−	Ach veh	8	84,50	2,50	15,70	3,00	7,80
CB1 +/+	Phe veh	7	192,00	12,70	855,00	276,00	6,07
CB1 −/−	Phe veh	8	196,10	11,20	1050,00	277,00	5,98
					EC50 umol/l	SE(EC50)	
CB1 +/+	Estradiol veh	10	18,20	1,90	5,05	N/A	5,30
CB1 −/−	Estradiol veh	10	25,90	2,10	1,88	N/A	5,73

LNA

genotype	treatment	N value	Emax%	SE(Emax%)	EC50 nmol/l	SE(EC50)	pEC50
CB1 +/+	Ach LNA	9	33,7	4,10	12,60	5,80	7,90
CB1 −/−	Ach LNA	9	30,2	4,10	12,60	N/A	7,90
CB1 +/+	Phe LNA	9	180,00	8,80	1190,00	264,00	5,92
CB1 −/−	Phe LNA	8	173,00	15,00	1060,00	431,00	5,97
					EC50 umol/l	SE(EC50)	
CB1 +/+	Estradiol LNA	10	5,56	2,50	N/A	N/A	N/A
CB1 −/−	Estradiol LNA	9	7,40	3,20	5,07	N/A	5,30

INDO

genotype	treatment	N value	Emax%	SE(Emax%)	EC50 nmol/l	SE(EC50)	pEC50
CB1 +/+	Ach INDO	9	92,8	3,90	16,30	3,90	7,79
CB1 −/−	Ach INDO	8	85,7	3,90	17,90	4,80	7,75
CB1 +/+	Phe INDO	8	139,80	10,00	1510,00	710,00	5,82
CB1 −/−	Phe INDO	7	113,20	10,90	1520,00	850,00	5,82
					EC50 umol/l	SE(EC50)	
CB1 +/+	Estradiol INDO	10	24,30	4,50	5,06	N/A	5,30
CB1 −/−	Estradiol INDO	9	16,90	5,00	5,10	N/A	5,29

Reference:

1. Szenasi A.; Amrein K.; Czeiter E.; Szarka N.; Toth P.; Koller A. Molecular Pathomechanisms of Impaired Flow-Induced Constriction of Cerebral Arteries Following Traumatic Brain Injury: A Potential Impact on Cerebral Autoregulation. *Int J Mol Sc*i. **2021** *Jun; 22(12): 6624.* doi: 10.3390/ijms22126624
